# Recent Advances in Vitamin E TPGS-Based Organic Nanocarriers for Enhancing the Oral Bioavailability of Active Compounds: A Systematic Review

**DOI:** 10.3390/pharmaceutics17040485

**Published:** 2025-04-07

**Authors:** Chee Ning Wong, Siew-Keah Lee, Yang Mooi Lim, Shi-Bing Yang, Yik-Ling Chew, Ang-Lim Chua, Kai Bin Liew

**Affiliations:** 1 M. Kandiah Faculty of Medicine and Health Sciences, Universiti Tunku Abdul Rahman, Kajang 43000, Malaysia; ggning100@1utar.my (C.N.W.); ymlim@utar.edu.my (Y.M.L.); 2 Institute of Biomedical Sciences, Academia Sinica, Taipei 115201, Taiwan; sbyang@ibms.sinica.edu.tw; 3 Faculty of Pharmaceutical Sciences, UCSI University, Kuala Lumpur 56000, Malaysia; chewyl@ucsiuniversity.edu.my; 4 Faculty of Medicine, Universiti Teknologi MARA, Sungai Buloh 47000, Malaysia; anglim@uitm.edu.my; 5 Faculty of Pharmacy, University of Cyberjaya, Cyberjaya 63000, Malaysia

**Keywords:** TPGS, vitamin E, oral bioavailability, organic nanocarriers, drug delivery

## Abstract

**Background:** D-α-tocopheryl polyethylene glycol 1000 succinate (TPGS), an amphiphilic derivative of natural vitamin E, functions as both a drug efflux inhibitor and a protector against enzymatic degradation and has been widely incorporated into nano-formulations for drug design and delivery. **Objective:** This systematic review evaluates TPGS-based organic nanocarriers, emphasizing their potential to enhance bioavailability of active compounds which include drugs and phytochemicals, improve pharmacokinetic profiles, and optimize therapeutic outcomes, eventually overcoming the limitations of conventional oral active compounds delivery. **Search strategy:** Data collection was carried out by entering key terms (TPGS) AND (Micelle OR Liposome OR Nanoparticle OR Nanotube OR Dendrimer OR Niosome OR Nanosuspension OR Nanomicelle OR Nanocrystal OR Nanosphere OR Nanocapsule) AND (Oral Bioavailability) into the Scopus database. **Inclusion criteria:** Full-text articles published in English and relevant to TPGS, which featured organic materials, utilized an oral administration route, and included pharmacokinetic study, were included to the final review. **Data extraction and analysis:** Data selection was conducted by two review authors and subsequently approved by all other authors through a consensus process. The outcomes of the included studies were reviewed and categorized based on the types of nanocarriers. **Results:** An initial search of the database yielded 173 records. After screening by title and abstract, 52 full-text articles were analyzed. A total of 21 papers were excluded while 31 papers were used in this review. **Conclusions:** This review concludes that TPGS-based organic nanocarriers are able to enhance the bioavailability of various active compounds, including several phytochemicals, leveraging TPGS’s amphiphilic nature, inhibition of efflux transporters, protection against degradation, and stabilization properties. Despite using the same excipient, variability in particle size, zeta potential, and encapsulation efficiency among nanocarriers indicates the need for tailored formulations. A comprehensive approach involving the development and standardized comparison of diverse TPGS-incorporated active compound formulations is essential to identify the optimal TPGS-based nanocarrier for improving a particular active compound’s bioavailability.

## 1. Introduction

Oral drug administration remains the most preferred route due to its numerous advantages. It is convenient, non-invasive, and enhances patient compliance, especially for long-term therapies. Oral formulations are also cost-effective to manufacture, store, and distribute, with versatile dosage forms such as tablets, capsules, and suspensions allowing for a flexible and controlled drug release. Nevertheless, the primary challenge in oral drug delivery is poor bioavailability, largely attributed to the first-pass metabolism, which significantly reduces the amount of the active compound reaching systemic circulation. Additionally, factors such as enzymatic degradation in the gastrointestinal tract, low solubility, and limited permeability further hinder effective drug absorption [1].

In addition to these challenges, many active compounds are chemically unstable and are prone to degradation when exposed to factors like light, heat, moisture, or gastrointestinal fluids, leading to reduced efficacy and shelf life [2]. Poor water solubility further limits an active compound’s ability to dissolve in the gastrointestinal fluids, a critical step for effective intestinal absorption [3]. Collectively, these issues contribute to diminished bioavailability, which may be further compromised by poor permeability across the intestinal lining or extensive first-pass metabolism in the liver.

Nanocarriers have been explored for oral drug formulation since the late 20th century, with significant advancements occurring in the early 2000s. The concept of nanoscale drug delivery systems, including liposomes and polymeric nanoparticles, was introduced in the 1970s–1980s. However, their application in oral formulations gained momentum in the 1990s with the development of solid lipid nanoparticles (SLNs) and polymer-based nanocarriers [4]. Over the past two decades, innovations in nanotechnology, such as self-emulsifying drug delivery systems (SEDDSs) and micelles, have further improved the bioavailability of poorly soluble drugs, making nanocarriers a crucial tool in modern oral drug delivery [5,6,7]. A nanocarrier is a nanoscale delivery system designed to encapsulate, protect, and transport active compounds, such as drugs, peptides, or bioactive molecules, to enhance their stability, solubility, and bioavailability. These carriers typically range in size from 1 to 1000 nm and are engineered to improve drug absorption, control the release, and target specific sites in the body [8,9]. Table 1 provides a comparative overview of various nanocarriers, categorized into polymer-based and lipid-based systems, as well as nanocrystals and nanosuspensions [10,11,12].

**Figure 1:**
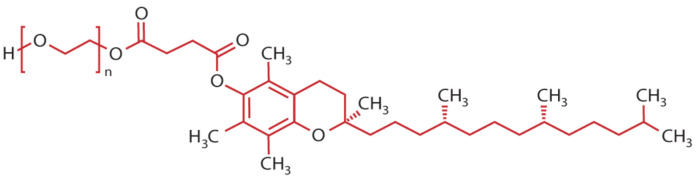
Chemical structure of D-α-tocopheryl polyethylene glycol 1000 succinate (TPGS). TPGS is a polymeric derivative of vitamin E, consisting of D-α-tocopheryl succinate conjugated with polyethylene glycol (PEG 1000). Its molecular weight is approximately 1513 Da, accounting for both the PEG 1000 segment and the tocopheryl succinate moiety.

Among these, D-α-tocopheryl polyethylene glycol 1000 succinate (TPGS) formulations have emerged as a promising solution. TPGS not only improves solubility and permeability but also enables sustained drug release, ultimately optimizing therapeutic efficacy and overcoming the inherent limitations of oral drug delivery [13,14]. TPGS is an amphiphilic derivative of natural vitamin E. synthesized through an esterification reaction between vitamin E succinate and polyethylene glycol (PEG) 1000. Its chemical structure consists of four key components: a chromanol ring, a phytyl tail, a succinate group, and a polyethylene glycol chain (Figure 1). The chromanol ring and phytyl tail form the foundational structure akin to that of vitamin E. Through an esterification process, the succinate group binds this structure to the PEG chain. TPGS is classified as a polymer derivative of vitamin E, having a molecular weight of approximately 1513 Da (with the PEG portion contributing around 1000 Da). It is typically a waxy solid at room temperature (25 °C) and liquefies upon slight heating (37 °C). This unique composition makes it particularly valuable in pharmaceutical and nutraceutical applications as an active compound carrier or excipient (Table 2).

The amphiphilic nature of TPGS, stemming from incorporating both hydrophilic polar heads and lipophilic alkyl tails, enables it to form hydrogen bonds with hydrophilic active compounds and engage in hydrophobic interaction with lipophilic active compounds. This dual interaction capability allows TPGS to effectively enhance the solubility and absorption of both hydrophilic and lipophilic drugs [15,16]. The increasing use of TPGS as an absorption enhancer is attributed to its unique combination of properties that overcome key limitations associated with other enhancers (Table 3) [17,18]. Unlike chitosan, which is limited by pH sensitivity and becomes less effective in the neutral to alkaline conditions of the intestine, TPGS remains effective across a broader pH range (4.5–7.5), fully adapting the intestinal environment. Compared to chelating agents, which may interfere with calcium-dependent processes and formulation stability, TPGS does not chelate essential ions, preserving physiological balance. In contrast to fatty acid derivatives, which are mainly effective for hydrophilic drugs and may cause irritation, TPGS is versatile, enhancing the absorption of both hydrophilic and lipophilic compounds due to its amphiphilic property. Moreover, TPGS is biodegradable, surfactant-like, and stabilizes nanoparticle formulations, offering protection to drugs against degradation while minimizing safety concerns. Although TPGS may face challenges, such as non-specific P-gp inhibition, this can be mitigated by incorporating specific ligands to enhance targeted delivery. This broad-spectrum capability, combined with its safety profile, positions TPGS as an outstanding and more versatile absorption enhancer in drug delivery systems.

Moreover, TPGS has been shown to disrupt the optimal functioning of P-glycoprotein (P-gp), a drug efflux transporter, by modulating membrane fluidity [19] and inhibiting P-gp adenosine triphosphatase (ATPase) activity [20]. Through these mechanisms, TPGS enhances the intracellular concentration and bioavailability of P-gp substrates. While TPGS has been widely recognized for its ability to enhance intracellular drug accumulation by inhibiting P-gp, it is crucial to acknowledge that indiscriminate P-gp inhibition in healthy tissues may lead to adverse effects. P-gp serves a protective role in physiological barriers such as the blood–brain barrier and intestinal epithelium, preventing the entry of xenobiotics and potentially harmful compounds. Uncontrolled inhibition could result in unintended toxicity and drug accumulation in non-targeted tissues [21]. To mitigate these risks, incorporating active targeting ligands into TPGS-based nanocarriers presents a promising approach to enhance their specificity toward diseased tissues, thereby improving therapeutic efficacy and reducing off-target effects. For instance, folic acid (FA) conjugation exploits the overexpression of folate receptors on certain cancer cells, facilitating receptor-mediated endocytosis of the drug-loaded nanocarriers. A study demonstrates that FA-modified TPGS micelles effectively deliver nitidine chloride to Huh7 human hepatocellular carcinoma cells, resulting in increased apoptosis compared to non-targeted formulations [22]. By enabling selective accumulation in pathological sites, such as tumor tissues, TPGS-based nanocarriers can effectively modulate P-gp activity where needed while minimizing systemic toxicity [23]. This targeted strategy not only enhances therapeutic efficacy but also reduces the potential risks associated with widespread P-gp inhibition.

Additionally, TPGS serves as a safeguard against enzymatic degradation for active compounds. Encapsulation of active compounds within TPGS coating effectively forms a protective shield around the active compound, blocking the access of degradative enzymes to the active pharmaceutical compound [24]. Consequently, this shielding effect minimizes interactions between active compounds and external biomolecules, thereby averting undesired chemical reactions. Additionally, storage-induced oxidative degradation could be eliminated by TPGS antioxidant capability, thereby stabilizing active compounds and preserving therapeutic effects [25].

TPGS is approved by the Food and Drug Administration (FDA) and the Japanese regulatory authorities as a pharmaceutical excipient, with the FDA allowing a maximum potency of 300 mg per unit dose [26,27]. In Europe and Canada, TPGS is approved for different applications; Europe permits its use in foods for special medical purposes at a maximum level of 58 mg per 100 g of product, while Canada recognizes it as a form of Vitamin E with a tolerable upper intake level of 1000 mg per day for adults [26,28,29]. TPGS can be incorporated into various product forms, including liquid formulations, capsules, and tablets administered via the oral route [14]. It is also used in topical, nasal, ophthalmic, and parenteral applications, providing versatility across different routes of administration [25].

TPGS has been approved for use in several specialized products in various countries, which includes the United States and in Europe. Notable examples include Ibuprofen (Boots, Nottingham, UK), Aptivus^®^ (Boehringer Ingelheim, Ingelheim, Germany), Agenerase^®^ (GlaxoSmithKline, London, UK), Viekira Pak™ (Abbvie, Chicago, IL, USA), Technivie™ (Abbvie, Chicago, IL, USA), Zepatier™ (Merck Sharp & Dohme, Rahway, NJ, USA), Fenofibrate (Abbott, Chicago, IL, USA), and Mavyret™ (Abbvie, Chicago, IL, USA), which incorporate TPGS in their formulations as a major inactive ingredient, serving as a solubilizer, surfactant, and therapeutic effect enhancer [14,30,31]. In contrast, Vedrop^®^ (Recordati Rare Diseases Ltd., London, UK) utilizes TPGS as an active ingredient for treating vitamin E deficiency caused by digestive disorders or conditions that impair fat absorption, particularly in children with malabsorption syndromes [32]. In addition to its role in active compound delivery, various studies have demonstrated that TPGS is a potent free-radical scavenger, which can be beneficial in combating oxidative stress [33]. This antioxidant feature makes TPGS particularly valuable in pharmaceutical and nutraceutical applications, serving not only as an active compound carrier or excipient but also as an adjunctive molecule that enhances therapeutic outcomes.

Various TPGS-based nanocarriers, each with unique structures and compositions, have been extensively explored for encapsulating a wide range of synthetic active compounds and molecules derived from natural sources. While inorganic nanocarriers offer excellent stability and controlled drug release, their potential for bioaccumulation raises safety concerns [34]. In contrast, organic nanocarriers are preferred for oral drug formulations due to their superior biocompatibility, biodegradability, and ability to enhance biocompatibility while minimizing toxicity risks [35]. Therefore, this review focuses on the role of TPGS-based organic nanocarriers as bioavailability enhancers, emphasizing their potential to improve the pharmacokinetic profiles and therapeutic outcomes of orally administered active compounds.

## 2. Method

### 2.1. Search Strategy

This review adhered to the preferred reporting items for systematic reviews and meta-analyses (PRISMA) guidelines and was registered in PROSPERO (CRD420251013140). The search was conducted using the search terms (TPGS) AND (Micelle OR Liposome OR Nanoparticle OR Nanotube OR Dendrimer OR Niosome OR Nanosuspension OR Nanomicelle OR Nanocrystal OR Nanosphere OR Nanocapsule) AND (Oral Bioavailability) in the Scopus database. The search was limited to articles published between 2019 and 2024, focusing on the document type. The last search was conducted on 15 February 2025.

### 2.2. Eligibility Criteria

Full-text articles published in English and relevant to TPGS, which featured organic materials, utilized an oral administration route, and included pharmacokinetic study, were included in the final review. The exclusion criteria include non-English articles, studies without accessible full texts, research focused on non-organic materials, studies unrelated to TPGS-based organic nanocarriers, those utilizing non-oral administration routes, and those lacking pharmacokinetic analysis.

### 2.3. Data Extraction and Variables of Interest

**Figure 2:**
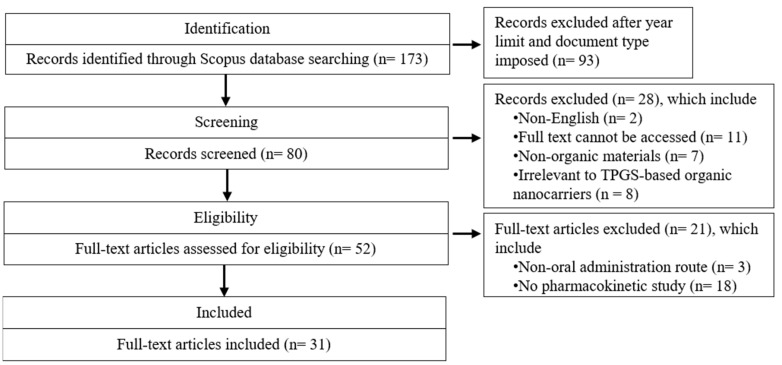
PRISMA Flowchart of study selection. TPGS: D-α-tocopheryl polyethylene glycol 1000 succinate.

An initial search of the database yielded 173 records. After screening by title and abstract, 52 full-text articles were analyzed. A total of 21 papers were excluded while 31 papers were included in the final review and analysis (Figure 2). Data selection was conducted by two review authors and subsequently approved by all other authors through a consensus process. The extracted data were further categorized based on types of nanocarriers, specifically polymer-based, lipid-based systems, nanocrystals, and nanosuspensions. Polymer-based nanocarriers include micelle, polymersome, and amorphous solid dispersion, while lipid-based systems encompass liposome, polymer lipid hybrid nanoparticle, niosome, solid lipid nanoparticle, lipid nanocapsule, and self-emulsifying drug delivery system. These classifications, along with key study variables, are summarized in Table 4. The analyzed variables include the type of nanocarrier, co-ingredient, preparation method, active compound, particle size, zeta potential, polydispersity index, encapsulation efficiency, and storage stability. Additionally, details on the experimental models and dosages used for pharmacokinetic studies, along with pharmacokinetic parameters such as area under curve (AUC), half-life (T_1/2_), and time to peak drug concentration (T_max_), are also presented in Table 4.

## 3. Main Content

This paper explores various nanocarrier systems incorporating TPGS for the formulation of active compounds, demonstrating four types of nanocarriers, namely polymer-based, lipid-based, nanocrystals, and nanosuspensions. Each nanocarrier is examined for its unique structural features and advantages in active compound encapsulation. The studies highlight TPGS’s potential for improving the stability and bioavailability of active compounds, providing a comprehensive overview of the current advancements in oral TPGS-based organic nanoparticle formulations. The key findings from the included studies are categorized based on the type of nanocarrier, including polymer-based (micelle, polymersome, amorphous solid dispersion), and lipid-based (liposome, polymer lipid hybrid nanoparticle, niosome, solid lipid nanoparticle, lipid nanocapsule, self-emulsifying drug delivery system), as well as nanocrystals and nanosuspensions (Table 4).

### 3.1. Polymer-Based Nanocarrier and TPGS

TPGS is considered a polymer due to its repeating ethylene-glycol units. It possesses several key qualities, including (i) amphiphilicity, (ii) inherent biocompatibility and biodegradability, (iii) drug efflux inhibition, and (iv) easy customization. These attributes make TPGS an ideal candidate for polymer-based nanocarriers, providing versatile and effective solutions for targeted active compound delivery applications [14].

#### 3.1.1. Micelle and TPGS

Micelles are spherical structures formed by the self-assembly of amphiphilic molecules. They typically range in size from 5 to 200 nm. Upon reaching the critical micelle concentration (CMC) and critical micelle temperature (CMT), the amphiphilic polymers undergo self-assembly, with hydrophobic alkyl tails congregating inward and forming a core that can encapsulate hydrophobic drugs, while the hydrophilic polar heads face outward, interacting with the surrounding aqueous medium [67]. Micelles are typically prepared using methods such as thin-film hydration, solvent evaporation, dialysis, or direct dissolution techniques [68]. Due to their ability to enhance solubility and bioavailability, micelles—both single and mixed—are commonly employed as active compound carriers [69,70]. In the included studies (Table 4), micelles exhibit a particle size ranging from 12 to 193 nm, with a zeta potential between –32 and +34 mV, and an encapsulation efficiency of 79–99%.

Paclitaxel (PTX), an anticancer drug, has limited water solubility (<0.1 µg/mL) [71] which restricts its oral bioavailability to below 10% [72]. Two studies have developed PTX-loaded mixed TPGS micelles with carboxymethyl chitosan and rhein [36], as well as with gallic acid and chitosan [43]. These micelles exhibit sustained release behavior when compared to Taxol (marketed PTX formulation), which is attributed to a strong hydrophobic interaction between PTX and the micellar core. This prevents drug precipitation during transport and ensures delivery to the target site. The enhanced oral bioavailability of PTX, as shown by augmented AUC and maximum concentration (C_max_) in experimental Sprague Dawley rats, along with TPGS-mediated P-gp efflux inhibition, contributes to an improved anti-tumor effect in a H22 liver tumor xenograft and a A549 lung tumor xenograft mice model.

Darunavir (DRV), an antiretroviral drug, exhibits low oral bioavailability due to its role as a P-gp efflux substrate. A solvent emulsification–evaporation method is employed to formulate DRV into poly(ε-caprolactone)/TPGS nanoparticles (DRV NPs) [37]. TPGS serves as both a P-gp inhibitor and stabilizer, preventing nanoparticle aggregation that could affect physical and chemical properties. DRV NPs exhibit a two-phase release profile; an initial, elevated release within 12 h, followed by a sustained release for up to 72 h. Reduced hepatic clearance and increased mean residence time (MRT) lead to prolonged plasma circulation, enhancing the oral bioavailability of DRV NPs in Sprague Dawley rats.

Due to its poor aqueous solubility and permeability, curcumin has been formulated in both single TPGS micelles [45] and mixed TPGS micelles combined with galactosamine, polyethylene glycol, and polylactic acid [38]. Encapsulation of curcumin within mixed micelles preserves its structural integrity and stability, facilitating a sustained release in release media. The incorporation of TPGS into a micelle enhances the systemic absorption and pharmacokinetics of curcumin, improving bioavailability after oral administration in Wistar rats and increasing the cytotoxic efficacy against HT-29 human colon cancer cells.

Zingerone-loaded TPGS mixed micelles (ZTMs) are formulated with phospholipid, sodium cholate, and PVP K30, employing a thin-film dispersion method. The successful encapsulation of zingerone into a micelle enhances its movement across the gastrointestinal mucosa, leading to a higher cumulative release in the dissolution media compared to the free form. Drug efflux inhibition by TPGS also significantly improves the uptake of the mixed micelle into human hepatocellular carcinoma (HepG2) cells, enhancing the anticancer effects, while also increasing the bioavailability of zingerone in Sprague Dawley rats [39].

Lopinavir, a human immunodeficiency virus (HIV-1) protease inhibitor, exhibits poor solubility and undergoes efflux-mediated first-pass metabolism, resulting in limited oral bioavailability. The incorporation of lopinavir into a micelle improves its dissolution and cumulative drug release. TPGS enhances lopinavir absorption by acting as (i) a stabilizer, (ii) a P-gp inhibitor, (iii) a metabolism modulator, (iv) a particle size reducer, and (v) a solubility enhancer, collectively facilitating lopinavir’s oral bioavailability in New Zealand rabbits [40].

Glycyrrhizic acid (GL) has been loaded into a pluronic F127/TPGS mixed micelle (F127/TPGS-MM) to maximize its clinical application as a hepatoprotective agent. The sustained release of GL from mixed micelles preserves structural integrity and prevents rapid precipitation within the gastrointestinal tract, ultimately enhancing its oral absorption. The improved bioavailability of GL, as substantiated by pharmacokinetic and tissue distribution profiles in Sprague Dawley rats, highlights the roles of F127 and TPGS as absorption enhancers that increase the cellular permeability of GL [41].

The poor bioavailability and dose-related side effects of aripiprazole (ARP) necessitate its development into a Soluplus/TPGS/borneol mixed micelle for improved therapeutic efficacy. The hydrophobic interaction between ARP and the micellar core leads to a gradual release of ARP from the mixed micelle. Additionally, the mixed micelle enhances the passive permeation of ARP across simulated intestinal and blood–brain barriers while reducing its efflux ratio, indicating TPGS-mediated P-gp inhibition. Ultimately, the mixed micelle improves the bioavailability of ARP, as evidenced by an increased AUC and C_max_ in CD1 mice [42].

TPGS has been combined with poly(ethylene glycol)-poly(ε-caprolactone) to formulate 6-gingerol into a mixed micelle. The small particle size and water-solubilizing effect of the TPGS mixed micelle enhance the plasma retention time of 6-gingerol, improving its oral bioavailability and tissue distribution in Sprague Dawley rats and Kunming mice. Notably, the concentration of 6-gingerol in the brain is significantly higher following administration of the micelle formulation, suggesting that TPGS effectively inhibits P-gp-mediated efflux and facilitates blood–brain barrier transport. This mechanism may potentiate the neuroprotective effect of 6-gingerol, positioning it as a promising brain-targeting therapeutic agent [44].

A myricetrin-loaded micelle with TPGS (MLTM) demonstrates improved solubility and stability, leading to higher cumulative diffusion, bioavailability, and tissue distribution compared to pure myricetrin in Sprague Dawley rats and Kunming mice [46]. Similarly, hesperetin-TPGS micelle, developed using the solvent dispersion method, significantly improves its water solubility and reduces the intestinal degradation and efflux through P-gp inhibition. This results in a 16.2-fold increase in the AUC in Sprague Dawley rats and improved hesperetin’s antioxidant activity in HepG2 cells [47].

#### 3.1.2. Polymersome and TPGS

Polymersomes are vesicular nanocarriers composed of amphiphilic block copolymers, typically ranging from 10 nm to 100 µm in diameter. They consist of a hydrophilic core and a hydrophobic bilayer membrane, allowing for the encapsulation of both hydrophilic and hydrophobic active compounds. Polymersomes are typically prepared using methods such as solvent switching, film rehydration, or electroformation techniques [73]. The functionalization of its vesicular membrane with specific coatings or ligands holds promise for targeted delivery, thereby improving the therapeutic effects [74].

In this review, a folic acid-pluronic F127-polylactic acid/TPGS (FA-F127-PLA/TPGS) mixed polymersome has been developed as a paclitaxel-loaded nanocarrier via the dialysis method. It exhibits a particle size of around 108 nm and an encapsulation efficiency of approximately 15%. The incorporation of TPGS into the mixed polymersome promotes interaction between paclitaxel and the non-polar alkyl groups of TPGS, facilitating drug encapsulation within the lipophilic bilayer and preventing drug leakage into the external environment. Consequently, the mixed polymersome exhibits a slower cumulative drug release. FA-F127-PLA/TPGS mixed polymersome has demonstrated a significant enhancement in paclitaxel uptake by Caco-2 cells. This improvement is likely due to TPGS-mediated inhibition of P-gp efflux activity, which prevents paclitaxel from being expelled into the extracellular space. As a result, the TPGS-based polymersome retains a higher intracellular concentration of the active compound, further enhancing the therapeutic effects. Moreover, FA-F127-PLA/TPGS mixed polymersome substantially improves the oral bioavailability of paclitaxel compared to FA-F127-PLA polymersome in Sprague Dawley rats, suggesting that TPGS enhances inhibition of both the cytochrome p-450 enzyme and P-gp transporter [48].

#### 3.1.3. Amorphous Solid Dispersion and TPGS

Amorphous solid dispersions refer to systems where poorly water-soluble crystalline active pharmaceutical ingredients (APIs) are dispersed within an excipient matrix, typically within the nanometer range. Amorphous solid dispersions improve drug solubility and bioavailability by converting the crystalline drug into a higher-energy, non-crystalline (amorphous) form, which exhibits greater dissolution rates than its crystalline counterpart. However, the amorphous form is thermodynamically unstable and susceptible to recrystallization. To overcome this limitation, stabilizing polymers such as polyvinylpyrrolidone (PVP), hydroxypropyl methylcellulose (HPMC), and methacrylate copolymers are incorporated to prevent crystallization and enhance the dispersion’s physical stability [75]. Amorphous solid dispersions typically appear as solid powders or films and require additional formulation techniques (e.g., spray-drying, hot-melt extrusion) for their manufacture [76].

A research group developed a curcumin (CUR)-loaded Kollidon CLSF amorphous solid dispersion, incorporating TPGS as a coating material. The addition of TPGS increased the curcumin solubility by 297-fold, demonstrating a significant solubility enhancement. Furthermore, encapsulating curcumin-loaded solid dispersion particles within the TPGS surfactant layer inhibits precipitation and recrystallization, thereby maintaining dispersion stability, as evidenced by (i) a prolonged nucleation induction time, (ii) a reduced crystal growth rate, and (iii) the absence of visible nucleation. The excellent cumulative drug release and moisture sorption of CUR/CLSF/TPGS suggest that TPGS facilitates strong hydrogen bonding interactions between the active compound and the nanocarrier, thereby improving storage stability. Consequently, the oral bioavailability of CUR/CLSF/TPGS increases by 1.28-fold, leading to a higher ulcer inhibition rate and enhanced healing effects in Sprague Dawley rats [49] (Table 4).

### 3.2. Lipid-Based Nanocarrier and TPGS

Lipid-based nanocarrier generally consist of lipid molecules such as phospholipids, cholesterol, and triglycerides, which form uniform lipid layers or solid cores to encapsulate active compounds [4]. TPGS serves as both a surfactant and emulsifier, making it well-suited for use in lipid-based nanocarriers due to its amphiphilic nature and ability to reduce interfacial tension.

#### 3.2.1. Liposome and TPGS

Liposomes are spherical vesicles characterized by a phospholipid bilayer encapsulating an aqueous core, with sizes typically ranging from twenty nm to several micrometers, depending on the preparation method. Common methods for preparing liposomes include thin-film hydration, reverse-phase evaporation, and ethanol injection [77]. The primary components of liposomes are phospholipids, such as phosphatidylcholine and cholesterol, which stabilize the bilayer structure [78]. They can encapsulate both hydrophilic drugs (within the aqueous core) and hydrophobic drugs (within the lipid bilayer) upon self-assembly in an aqueous medium [79]. Liposomes can be stabilized through steric stabilization with polyethylene glycol [80], incorporation of a silicone network within the bilayer [81], and the use of charged nanoparticles [82], which enhance their stability and functionality for active compound delivery. The liposomes reported in the included studies (Table 4) display a particle size within the range of 23–190 nm, a zeta potential from –45 to –19 mV, and an encapsulation efficiency between 86% and 96%.

Bisdemethoxycurcumin (BDMC), a curcuminoid derived from *Curcuma longa*, exhibits greater stability and resistance to alkaline degradation than curcumin. Like curcumin, BDMC has limited solubility and bioavailability, which restricts its clinical applications. To overcome these challenges, a BDMC-conjugated TPGS liposome has been developed as a nanocarrier to enhance the efficacy of BDMC. The cumulative release profile of BDMC-TPGS liposome shows a significantly higher release rate than free BDMC, likely due to TPGS which functions as an emulsifier, stabilizer, and solubilizer. This improves drug diffusion from nanoparticles, promoting effective analgesic and anti-hyperuricemic effects in Sprague Dawley rats. Additionally, the liposomal formulation markedly enhances the oral bioavailability of BDMC in Sprague Dawley rats by inhibiting P-gp efflux and providing protection against degradation through TPGS incorporation [50].

The thin-film dispersion method is used to formulate TPGS-coated 6-shogaol liposome, incorporating phospholipid, cholesterol, sodium cholate, and isopropyl myristate as key components. The higher cumulative drug release rate observed in TPGS-coated 6-shogaol liposome suggests that TPGS enhances 6-shogaol solubility and promotes membrane diffusion compared to uncoated liposome. Additionally, the TPGS coating inhibits drug efflux and reduces recognition by the reticuloendothelial system (RES), as evidenced by lower liver accumulation, thereby prolonging systemic circulation and enhancing bioavailability in Sprague Dawley rats [51].

A rapamycin-loaded TPGS-lecithin-zein nanoparticle (RTLZ-NP) is prepared using the phase separation method to enhance rapamycin’s oral bioavailability. Rapamycin is highly sensitive to acidic environments and is prone to enzymatic degradation, resulting in reduced retention in simulated gastric fluids (SGFs) and simulated intestinal fluids (SIFs). TPGS-coated rapamycin liposome retains 69% and 94% of the active compound after 2 h in SGFs and SIFs, respectively, compared to 43% and 91% for non-TPGS-coated liposome, demonstrating enhanced resistance to enzymatic breakdown. Additionally, TPGS acts as a P-gp inhibitor, enhancing intracellular uptake across Caco-2 cells by increasing influx permeability and reducing efflux permeability. The high retention and slow clearance of RTLZ-NP contribute to improved pharmacokinetics, reflected by a greater AUC and C_max_ in Sprague Dawley rats [52].

To address the poor aqueous solubility and rapid elimination of syringic acid, a syringic acid-loaded TPGS liposome (SA-TPGS-L) has been developed. This formulation increases the AUC by 2.8-fold compared to pure syringic acid in Sprague Dawley rats, which is attributed to TPGS’s role in reducing gastrointestinal degradation and enhancing cellular permeation. Additionally, SA-TPGS-L achieves broader tissue distribution and delayed clearance, thereby improving its in vivo antioxidant activity in Kunming mice [53].

#### 3.2.2. Polymer–Lipid Hybrid Nanoparticle and TPGS

A polymer–lipid hybrid nanoparticle was first conceptualized as the combination of polymer- and lipid-based nanoparticles, typically ranging from 60 nm to 400 nm in diameter. It consists of a polymeric core encapsulated within a lipid layer. This dual composition allows the polymer–lipid hybrid nanoparticle to inherit the advantages of both parent carriers, including small size and high active compound loading [83]. A polymer–lipid hybrid nanoparticle can be prepared using one-step or two-step methods. The two-step method is commonly used in early formulation and involves forming lipid vesicles and polymeric nanoparticles separately, then combining them via electrostatic interactions. However, the two-step process is time-consuming and energy-intensive, prompting the development of a simpler one-step method, in which lipid and polymer solutions are combined in a single step using nanoprecipitation or emulsification-solvent evaporation [84]. Polymer–lipid hybrid nanoparticles reported in the included studies (Table 4) exhibit particle sizes ranging from 136 to 192 nm, with an encapsulation efficiency between 88% and 92%; however, the zeta potential is assessed in only one study, showing a value of −35 mV.

A study has developed a TPGS-based polymer–lipid hybrid nanoparticle (TPGS-PLHNP) using exemestane (EXE) as the hydrophobic model active compound, forming EXE-TPGS-PLHNP. The enhanced delivery efficiency of the active compound is attributed to the inhibitory action of TPGS on the efflux transporter P-gp. Incorporating TPGS into EXE-PLHNP results in approximately 30% and 28% improvements in AUC and C_max_, respectively, due to (i) enhanced EXE solubilization, (ii) controlled EXE release, and (iii) augmented intestinal permeation and absorption, all contributing to improved oral bioavailability of EXE in Wistar rats. The safety and biocompatibility of EXE-TPGS-PLHNP are evaluated in a toxicity study on Wistar rats, with no significant differences observed in biochemical parameters and histopathological examinations between the control and formulation-treated groups [54].

Another research group modifies TPGS with poly(methyl vinyl ether-co-maleic anhydride) to develop a novel PVMMA-TPGS copolymer, and incorporates it into the polylactic-co-glycolic acid (PLGA)/lipid hybrid nanoparticle (PLGA NP) to load cabazitaxel (CTX), resulting in the formation of CTX-PTNP. The slow and sustained release profile, along with strong intestinal retention, lead to the improved bioadhesion of CTX-PTNP. As a result, CTX-PTNP displays higher cumulative permeation across the small intestine, owing to the mucoadhesive property of PVMMA and the P-gp inhibition effect of TPGS. These factors—(i) high drug entrapment stability, (ii) improved intestinal retention and mucoadhesion, and (iii) augmented intestinal absorption—ultimately contribute to the increased oral bioavailability of CTX in Sprague Dawley rats [55].

#### 3.2.3. Niosome and TPGS

Niosomes are spherical, lamellar structures composed of non-ionic surfactants and cholesterol, typically ranging from 10 nm to 5 µm in size. They share structural similarities with liposomes but differ in their constituent materials. Niosomes can encapsulate both hydrophilic and hydrophobic drugs, offering advantages such as improved stability and cost-effectiveness over liposomes [85,86]. Traditional niosome preparation methods, such as thin-film hydration and reverse-phase evaporation, are limited by poor particle size control and low encapsulation efficiency, leading to the development of newer techniques like microfluidics and ball milling for better control. Alternatively, optimization methods like sonication or extrusion are required to enhance size control and distribution [87].

Sulphonyl-guanidine-containing pyrazolopyrimidine has been derived as a vascular endothelial growth factor receptor (VEGFR) inhibitor, an anticancer agent. However, its poor aqueous solubility restricts its therapeutic efficacy, necessitating the development of a more orally bioavailable formulation. Hence, a research team formulated it into TPGS-coated niosome using the thin-film hydration method. The niosome demonstrates a particle size of 138 nm, a zeta potential of −45 mV, and a high encapsulation efficiency of 90% (Table 4). In vitro drug release study reveals a higher percentage of drug release from the TPGS-coated niosomal formulation compared to the free suspension, likely due to the enhanced solubilization afforded by the hydrophilic nature of TPGS. The therapeutic effects of the TPGS-coated niosomal formulation are compared to the uncoated niosomal formulation, showing significant improvements in the following areas: (i) lower half maximal inhibitory concentration (IC_50_) against HepG2 cells (1.6 µM and 2 µM, respectively), (ii) greater inhibition on both vascular endothelial growth factor (VEGF) and VEGFR expressions (80.4% to 76% and 80% to 70%, respectively), and (iii) increased cytotoxicity towards VEGF expression. These results can be attributed to (i) the enhanced solubility and absorption of the active compound, (ii) the multidrug resistance inhibition, and (iii) the prolonged active compound circulation time within the body system, due to the incorporation of TPGS onto the niosome surface. An oral bioavailability study is conducted in Wistar albino rats comparing TPGS-modified niosome with the pure suspension, showing a 4.5-fold increase in the AUC and a 3.6-fold increase in the C_max_ [56].

#### 3.2.4. Solid Lipid Nanoparticle and TPGS

Solid lipid nanoparticles (SLNs) are spherical particles composed of a solid lipid core stabilized by a surfactant monolayer, with sizes typically ranging from 50 to 1000 nm. SLNs can be prepared through high-pressure homogenization, microemulsion, solvent emulsification–evaporation, and double emulsion methods [88]. The solid lipid core, made from long chain fatty acid derivatives [57,58], can encapsulate hydrophobic drugs, enhancing their stability and controlled release. SLNs combine the advantages of other nanocarriers, such as biocompatibility and improved active compound bioavailability, but face limitations such as a low drug loading capacity and drug expulsion during storage due to the rigidity of the highly ordered lipid matrix. To improve drug loading, the use of complex lipids is preferred over simple ones to create loosely packed crystals with greater imperfections. Emulsifying agents have been incorporated to enhance their stability [89]. A particle size ranging from 114 to 256 nm, along with a zeta potential between −12 and −10 mV, and an encapsulation efficiency of 72–84% are observed for the solid lipid nanoparticles in the included studies (Table 4).

A naringenin-loaded SLN (naringenin-SLN) has been developed using TPGS as the outer surfactant layer and glyceryl monooleate (GMO) as the solid lipid core. In the drug release study, it shows an initial burst release stage followed by a slow, controlled release. After 36 h of incubation, 79% of naringenin is released from the nanoparticle, in contrast to 96% of pure naringenin released within the first 6 h, indicating that TPGS protects naringenin from degradation. In addition, significant improvements are observed in the pharmacokinetic indices of naringenin-SLN compared to the pure active compound (AUC: 15 and 2 µg/mL × h; C_max_: 4.3 and 0.3 µg/mL). The enhanced solubility, prolonged circulation, and sustained drug release of naringenin-SLN are crucial to improve its oral bioavailability and antifibrotic effects in Wistar rats [57].

Asenapine maleate (AM), an antipsychotic active compound, is fabricated as a solid lipid nanoparticle using glycerol monostearate (GMS), TPGS, and poloxamer 188, employing a high-speed homogenization–ultrasonication method. For AM-SLN, the active compound must escape from both the inner lipid core and the outer TPGS/poloxamer layer before diffusing into the release medium, resulting in a much slower and more sustained drug release profile. Oral administration of AM-SLN to Sprague Dawley rats reveals significant improvements in both the AUC and C_max_ compared to AM dispersion (50-fold and 20-fold increase, respectively). This improved oral bioavailability can be attributed to (i) a small particle size, which helps bypass first-pass hepatic metabolism, (ii) a larger surface area, which aids in the diffusion and absorption, and (iii) its mucoadhesive characteristic, which prolongs the retention of the active compound [58].

#### 3.2.5. Lipid Nanocapsule and TPGS

Lipid nanocapsules are vesicular nano-sized carriers (20–100 nm), combining features of polymeric nanocapsules and liposomes. They consist of a lipid core, typically made of medium-chain triglycerides, surrounded by a surfactant shell composed of PEGylated surfactants and, optionally, lecithin or other co-surfactants. These nanocapsules are capable of encapsulating hydrophobic drugs within their lipid core, offering advantages such as improved drug stability and controlled release. They are typically prepared using nanoprecipitation and phase-inversion techniques, and offer enhanced stability as compared to liposomes [90,91,92].

TPGS has been selected as the stabilizer for curcumin-loaded lipid nanocapsules. These nanocapsules exhibit a particle size of 190 nm, with an encapsulation efficiency of 51%. They exhibit high stability in both gastrointestinal environments and storage conditions (4 °C and 25 °C), likely due to the TPGS modification, which introduces electrical charges on the nanocapsule surface, preventing undesired particle aggregation. Native curcumin undergoes rapid drug release within 12 h, leading to a reduced circulation time and accelerated drug elimination. However, when encapsulated within lipid nanocapsules, the drug release profile changes, showing an initial burst followed by sustained and controlled release. Furthermore, curcumin loaded into TPGS-coated nanocapsules results in a 5.6- and 12.3-fold increase in the C_max_ and AUC in Sprague Dawley rats, implying the potential of TPGS in enhancing active compound absorption and intestinal permeability [59] (Table 4).

#### 3.2.6. Self-Emulsifying Drug Delivery System and TPGS

The self-emulsifying drug delivery system (SEDDS) is an isotropic mixture of oils, surfactants, and, occasionally, co-surfactants, which forms fine oil-in-water emulsions or microemulsions upon dilution in aqueous media [93]. Upon formation, the active compound interacts with the oil, creating a lipidic solution. The addition of a surfactant and, if applicable, a co-surfactant, acts as an emulsifying agent by (i) lowering the interfacial tension between dispersed lipid molecules and the aqueous phase, (ii) forming a protective layer around each lipid molecule, and (iii) preventing coalescence of lipid droplets, thereby stabilizing the emulsion system [94,95]. SEDDS can be further classified based on droplet size to (i) self-nanoemulsifying drug delivery system (SNEDDS), with droplets smaller than 100 nm, and (ii) self-microemulsifying drug delivery system (SMEDDS), with droplet sizes ranging from 100 to 250 nm [96]. SEDDS enhance the oral bioavailability of poorly water-soluble drugs by facilitating their solubilization and absorption in the gastrointestinal tract. The SEDDS formulations in the included studies (Table 4) demonstrate a particle size ranging from 30 to 320 nm, with zeta potential values varying between −28 mV and +17 mV. Among them, only one study assesses encapsulation efficiency, which is found to be 95%.

The low bioavailability of quercetin (QU) has sparked research interest in developing a TPGS-based SNEDDS formulation. Pharmacokinetic studies in Wistar rats have demonstrated a significant improvement in QU-SNEDDS compared to QU suspension, as indicated by a higher C_max_ (491.3 µg/L vs. 163.2 µg/L) and AUC (2286 µg/L × h vs. 1525.7 µg/L × h). Overall, the bioavailability of QU following oral administration increases by 149.8%, attributed to (i) enhanced QU permeability across the gut membrane, (ii) increased surface area due to small globule size, improving QU absorption, and (iii) a stabilized formulation preventing QU degradation during delivery [60].

The QU-SNEDDS formulation discussed earlier exists in a liquid state, commonly referred to as liquid SEDDS. However, this form presents challenges such as chemical instability, limited dosage form options, and the risk of active compound precipitation. These limitations can be addressed by converting liquid SEDDS into solid SEDDS through a solidification process [96].

The two formulations discussed below are solid SEDDS. In the first study, the poorly soluble oral anticoagulant, rivaroxaban, is developed into a SNEDDS using high-pressure homogenization emulsification and solidified with calcium silicate. Upon oral intake, the solid SNEDDS interacts favorably with gastrointestinal fluids, forming an oil-in-water nanoemulsion that facilitates the dissolution of the active compound in a supersaturated state. As expected, oral administration of 0.5 mg/kg rivaroxaban-loaded solid SNEDDS in Sprague Dawley rats results in a significantly higher plasma concentration of rivaroxaban compared to rats treated with pure rivaroxaban powder, highlighting the role of TPGS as a solubility and permeation enhancer [61].

In the second study, a silica-based paclitaxel-loaded solid SEDDS (Si-PTX-S-SEDDS) is formulated. The spray-drying method is used to solidify the SEDDS with fumed colloidal silica as the solidifying agent. TPGS is incorporated into the oil phase due to its ability to inhibit the P-gp efflux transporter and its synergistic anticancer effect with paclitaxel. An in vitro release study is conducted on both Taxol (the marketed paclitaxel formulation) and Si-PTX-S-SEDDS. After 24 h of incubation in dissolution media, Taxol exhibits a higher paclitaxel release (53%) than Si-PTX-S-SEDDS (32%). Encapsulation of paclitaxel within the nanoemulsion core preserves its integrity and restricts its dissolution into the surrounding media. Consequently, the oil-phase components and surfactant coating in PTX-SEDDS enable a controlled and sustained drug release, thereby extending the circulation time of the active compound and enhancing therapeutic outcomes. The oral bioavailability of paclitaxel via Si-PTX-S-SEDDS administration (21.32%) is approximately five times higher than that of Taxol (4.28%) in Sprague Dawley rats [62].

### 3.3. Nanocrystals and TPGS

Nanocrystals are crystalline particles of the active pharmaceutical ingredient, typically in the nanometer size range [97]. They enhance the solubility and dissolution rate of poorly water-soluble drugs by increasing the surface area available for dissolution. Nanocrystals can be prepared using top-down approaches, such as milling, or bottom-up approaches, such as precipitation. Composed entirely of drug material, nanocrystals can passively target tumors via the enhanced permeability and retention effect but face challenges like poor targeting efficiency and uncontrolled drug release. To overcome these issues, surface modifications with ligands and stabilizers are used to enhance targeting, circulation time, and stability [98]. The database search identified only two studies related to nanocrystals, both conducted by the same research team. In these studies, andrographolide (ADR) is used as the model active compound, with differences in the formulation ingredients used to develop the nanocrystals. Their particle size ranges from 573 to 604 nm, with one study reporting a zeta potential of +46 mV.

The research team initially developed two formulations: stabilizer-free andrographolide nanocrystal (AN) and TPGS-modified andrographolide nanocrystal (TAN). X-ray diffraction (XRD) analysis shows the absence of ADR’s characteristic peaks in TAN, suggesting a transition from a crystalline to an amorphous state, which could enhance drug dissolution. The saturation solubility of ADR in water is 40.79 µg/mL, while surface modification with TPGS increases it to 65.34 µg/mL, highlighting the solubilizing effect of the hydrophilic PEG moiety in TPGS. The incorporation of TPGS significantly improves ADR bioavailability in Sprague Dawley rats, likely due to its solubilizing properties and P-gp inhibition effects, both of which enhance active compound absorption [64].

The team further explores the conjugation of TPGS with sodium dodecyl sulfate (SDS), forming the SDS-TPGS copolymer, which is used to develop SDS-TPGS-modified andrographolide nanocrystal (S-TAN). Similarly to TAN, S-TAN lacks ADR’s characteristic peaks, indicating that SDS-TPGS integration alters ADR crystallinity. The pharmacokinetic studies conducted in Sprague Dawley rats show that the AUC and C_max_ values for TAN are 2.677 mg/L × h and 0.235 mg/L, respectively, while those for S-TAN increase to 3.832 mg/L × h and 0.602 mg/L. This improvement is attributed to SDS-enhanced paracellular permeability, facilitating ADR internalization [63]. This study highlights the potential of combining TPGS with other stabilizers to improve active compound bioavailability. While such a conjugation may enhance drug delivery and therapeutic outcomes, it also presents the potential drawback of increased particle size, requiring careful evaluation.

### 3.4. Nanosuspensions and TPGS

Nanosuspensions are submicron colloidal dispersions of pure drug particles stabilized by surfactants, with particle sizes typically below 1 μm [99]. The preparation methods for nanosuspensions can be classified into two main categories: the bottom-up and top-down approaches. The bottom-up method involves the self-assembly of particles from a molecular state to nanoscale size, while the top-down method entails the reduction of larger particles into nanoparticles. Several post-production processes, such as freeze-drying, spray-drying, and spray-freezing, can be employed to transform a nanosuspension into different dosage forms (e.g., powders, capsules, and films) while preserving its stability [99,100]. The particle size of nanocrystals in Table 4 ranges from 182 to 233 nm, with one study reporting a zeta potential of −8 mV.

A quercetin-loaded nanosuspension (Que-NSp) is prepared using TPGS as a stabilizer via the tetragonal zirconia polycrystal (TZP) grinding method, a top-down technique. The TPGS-stabilized Que-NSp exhibits three distinct release phases: (i) an initial rapid burst release within the first 30 min, (ii) a controlled release phase lasting up to 8 h, and (iii) a subsequent slower release phase that plateaus at 24 h. However, this study does not analyze the drug release behavior of pure quercetin, preventing direct comparisons and limiting the ability to draw meaningful conclusions. This represents a key limitation of the research. The oral bioavailability of quercetin in Sprague Dawley rats is enhanced due to a (i) higher C_max_, (ii) a prolonged time to reach C_max_, and (iii) a greater AUC. The role of TPGS as a surfactant contributes to several key effects, such as (i) facilitating the uptake of nanosuspension particles into the gastrointestinal tract by increasing the affinity between lipid particles and the intestinal membrane, (ii) inhibiting P-gp activity, thereby reducing drug efflux and extending retention time, (iii) reducing particle size due to steric hindrance between nanosuspension surfaces, leading to the circumvention of the hepatic first-pass metabolism, and (iv) minimizing quercetin metabolism and degradation, thereby prolonging its residence time in systemic circulation [65].

The following study investigates the lipophilic drug ticagrelor (TCG) as a model. Preliminary screening identifies TPGS and polyvinyl alcohol (PVA) as the optimal surfactant and polymer, respectively, due to TCG’s highest solubility when in TPGS and the smallest particle size achieved when combined with PVA. The optimized ticagrelor-dispersed nanosuspension (TCG-NSP) significantly improves TCG dissolution across various media (pH 1.2, pH 4.0, pH 6.8, and distilled water) compared to Brilinta^®^ (AstraZeneca Pharmaceuticals LP, Wilmington, DE, USA) (a commercially available TCG formulation) and the physical mixture. This enhancement is attributed to (i) improved wettability and localized solubilization within the diffusion layer, (ii) transformation of the crystalline drug form into an amorphous state, and (iii) drug supersaturation facilitated by a reduced particle size and increased surface area. The higher apparent permeability coefficient of TCG-NSP compared to pure TCG and Brilinta^®^ may be due to the entanglement of TCG particles with the mucin layer and the inhibitory effect of TPGS on P-gp efflux, leading to enhanced intestinal permeation and increased intracellular drug concentration. As expected, the pharmacokinetic findings align with previous observations (enhanced solubility and permeability), confirming a notable increase in TCG bioavailability following oral administration in Sprague Dawley rats. TPGS plays a crucial role in boosting paracellular drug absorption and protecting against drug metabolism by degradative enzymes in the gastrointestinal tract [66].

## 4. Discussion

**Figure 3:**
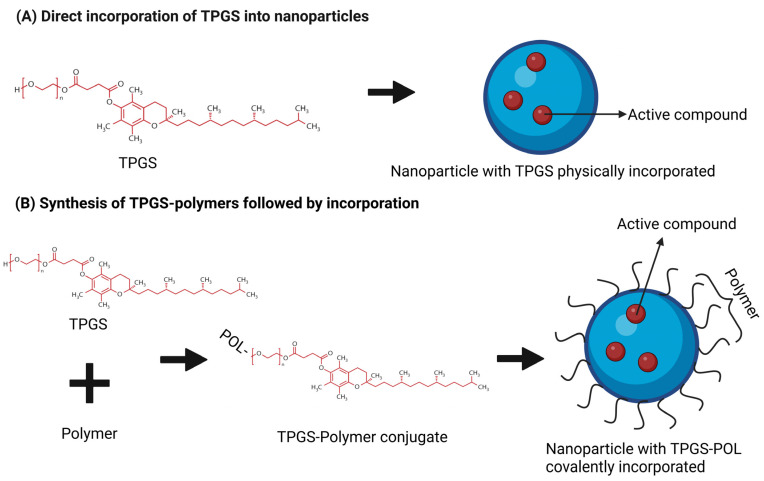
Strategies for incorporating TPGS into nanoparticles: direct incorporation vs. covalent conjugation.

The incorporation of TPGS into nanoparticles can be achieved through two distinct strategies: direct physical incorporation and covalent conjugation followed by incorporation (Figure 3). In the direct approach, TPGS is physically blended with nanoparticle components, allowing its integration into the nanoparticle matrix via non-covalent interactions such as hydrophobic interactions and hydrogen bonding. This method is simple, cost-effective, and preserves the native bioactivity of TPGS; however, it may suffer from limitations such as the potential leaching of TPGS from the nanoparticles and reduced long-term stability [101]. In contrast, the second strategy involves the chemical synthesis of TPGS-polymer (TPGS-POL) conjugates, where the hydroxyl group at the PEG chain of TPGS is conjugated with a polymer before incorporation into nanoparticles. This covalent conjugation provides enhanced stability by anchoring TPGS within the nanoparticle matrix and allows greater control over nanoparticle properties, including drug release profiles and surface characteristics. Additionally, the conjugation of specific polymers can facilitate targeted delivery as these polymers can serve as ligands for receptors on target cells, enabling selective binding and thereby enhancing the specificity and efficiency of drug delivery [30]. Despite these advantages, this method is more complex and resource-intensive, and the chemical conjugation may alter some of TPGS’s inherent functionalities. Thus, the choice between these strategies depends on the specific application requirements, balancing simplicity and functionality against stability and customization [102].

Bioavailability, the extent and rate of drug permeation into systemic circulation, reflects the drug’s journey to the target site [103]. It can be influenced by several key physicochemical characteristics, including the particle size, polydispersity index, and zeta potential [104]. Each of these parameters plays a significant role in determining how efficiently the drug is absorbed, distributed, and metabolized in the gastrointestinal tract, thus impacting its overall therapeutic efficacy. A particle size within the 300 nm range is considered ideal for cellular uptake and internalization [105,106]. All the studies in Table 4 achieve this criterion, except for nanocrystal-based, which may be attributed to the addition of a large-sized excipient (besides TPGS). The relatively small size of nanocarriers can enhance drug absorption by facilitating cellular uptake and improving bioavailability. While the enhanced permeability and retention (EPR) effect is well-documented in the context of solid tumors, where leaky vasculature and impaired lymphatic drainage allow nanoparticles to accumulate preferentially in tumor tissues [107], its role in non-cancerous applications differs. In non-tumor physiological conditions, small-sized nanocarriers can still improve drug absorption through mechanisms such as transcellular transport and endocytosis, leading to increased bioavailability [108]. Moreover, the P-gp inhibitory activity of TPGS plays a crucial role in overcoming drug efflux mechanisms, which can further enhance the therapeutic efficacy of drug-loaded nanocarriers, particularly in cancer treatment where drug resistance is a challenge [14].

The polydispersity index (PDI) is a measurement of particle size distribution, ranging from 0 to 1. A monodisperse sample has a low PDI value while a high value (close to 1) indicates high heterogeneity within a sample. A PDI ≤ 0.2 is considered as an acceptable value in nanoparticle formulation [104]. A total of 54% of the studies (Table 4) have achieved the desired PDI value, suggesting the use of an optimal TPGS-containing formulation ratio and preparation method.

The electric charge on the particle surface is a crucial factor in determining particle stability, assessed through the parameter known as zeta potential. Optimal stability is linked with a zeta potential value surpassing +30 mV or falling below −30 mV [109]. A larger absolute zeta potential value enhances particle stability by increasing the repulsion between similarly charged particles. This enhanced stability prevents aggregation [110], which is especially important for the oral administration of drug nanoparticles. When nanoparticles aggregate, their increased size can hinder effective absorption in the gastrointestinal tract, reducing the drug’s therapeutic efficacy. Among the studies below (Table 4), only eight TPGS-based nanoparticles have successfully attained the target zeta potential (however, some studies did not assess this aspect), which can be ascribed to the presence of surfactant layers (other than TPGS) with more pronounced positive or negative charges in their composition. This is because the zeta potential largely depends on the materials used in the nanoparticle’s outer shell, where TPGS is commonly combined with other excipients in most studies.

Encapsulation efficiency refers to the proportion of the active compound successfully entrapped in a nanocarrier system. Several factors, including the concentration, solubility, and compatibility of polymers or surfactants, strongly influence the strength of interaction between the active compound and its carrier [111]. It is a key determinant of the dosage-dependent bioavailability of the encapsulated drug as higher encapsulation efficiency ensures a greater proportion of the active compound is delivered into systemic circulation. Among the studies reviewed (Table 4), a majority of the studies showed an encapsulation efficiency of >80%, with an increased AUC and C_max_. Interestingly, despite a relatively low encapsulation efficiency of approximately 15% reported in one study, the formulation exhibited a remarkable 6-fold increase in the AUC and a 4-fold increase in the C_max_, highlighting the potential of TPGS nanocarriers in optimizing drug absorption and systemic exposure. This low encapsulation efficiency may stem from weak interactions between paclitaxel and the encapsulating matrix or inefficient entrapment during formulation. While a higher encapsulation efficiency typically enhances bioavailability, other factors—such as the particle size, surface properties, release kinetics, and interactions with biological membranes—also play a crucial role. Therefore, the paradox of how low encapsulation efficiency yet improved bioavailability may be attributed to the conjugation of folate groups onto the polymersome. This modification likely enhances the interaction with folate receptors in the gastrointestinal tract, facilitating uptake and ultimately improving the bioavailability of this TPGS nanocarrier.

Storage stability is a critical factor in ensuring the quality, safety, and efficacy of pharmaceutical products by preventing degradation and maintaining therapeutic potency [112]. Stability assessments encompass two primary aspects: physical stability and chemical stability, both of which can be influenced by environmental factors such as temperature, air exposure, light, and humidity. Physical stability refers to the preservation of a product’s appearance, texture, and form, which can be affected by changes such as aggregation, sedimentation, or polymorphic transitions. These alterations may compromise the drug’s performance and bioavailability. On the other hand, chemical stability focuses on maintaining the integrity of the active pharmaceutical ingredient (API), preventing degradation processes such as oxidation, hydrolysis, or structural breakdown, which can lead to reduced efficacy or the formation of toxic byproducts [113]. While environmental factors contribute to both stability aspects, the underlying mechanisms differ, necessitating distinct evaluation strategies. Given the importance of long-term stability, regulatory guidelines such as ICH Q1A (R2) recommend a minimum of 12 months of stability testing under long-term storage conditions to establish an appropriate shelf life and ensure product reliability over time [114]. However, our literature survey indicates that many studies evaluate stability for short-term periods (0.25–6 months), which may not adequately capture long-term degradation trends. To bridge this gap, we emphasize the need for extended storage stability studies, particularly for nanoparticle-based formulations where parameters such as the particle size, zeta potential, and encapsulation efficiency must be monitored over time.

The toxicological assessment of a novel compound is vital in the drug development process. Species-, organ-, and dose-specific adverse effects of the active compound can be elucidated by preclinical toxicity evaluations conducted across various biological systems, aiding in determining the no observed adverse effect level (NOAEL) which is essential for initiating clinical investigations [115]. In this review paper, among the 31 studies examined, 13 conducted toxicity assessments, with only 2 focused on acute toxicity, while the rest examined cytotoxicity. The neglect of this aspect by researchers may indicate a lack of emphasis on safety and pose concealed risks for future pharmaceutical compound development. As previously emphasized, safety evaluations and toxicity profiling are crucial for the ensuring safe administration and a potential supplementation strategy in various pharmaceutical formulations and active compound delivery systems [116]. The ultimate goal of developing a novel drug is its eventual market approval as a therapeutic agent or dietary supplement [117]. Overlooking toxicity study, a critical component of the preclinical phase, can impede the active compound’s progression into clinical phases, hindering its potential development. Hence, it is imperative for researchers to prioritize and address this issue, ensuring the safety and success of drug development endeavors.

Even with the same type of model active compound (i.e., paclitaxel in studies 1, 8, 13, and 27; curcumin in studies 3, 10, 14, and 24), TPGS-based nanoparticles can result in different levels of bioavailability. The effectiveness of TPGS can be influenced by the concentration of the active compound, as higher or lower doses may impact the extent of drug release, absorption, and distribution. Additionally, variations in the additional excipients or polymers paired with TPGS can alter the stability, release profile, and interaction of the nanoparticles within biological systems. For instance, certain polymers may enhance the mucoadhesive properties of the nanoparticles, extending their residence time in the gastrointestinal tract and increasing absorption [118], while others may alter the nanoparticle size or zeta potential, further influencing bioavailability. Thus, even with the same core active compound, the specific formulation design—the dosage and ingredients—paired with TPGS can create notable differences in the resulting bioavailability, underscoring the importance of precise formulation optimization in TPGS-based drug delivery systems.

## 5. Conclusions and Future Perspectives

Numerous nanocarriers have been employed by researchers to develop TPGS-based formulations, aiming for the improved bioavailability of orally ingested active compounds. TPGS offers several advantages, such as (i) its amphiphilic nature enabling solubilization of both hydrophilic and lipophilic active compounds, (ii) its inhibition on efflux transporters, (iii) its protection against degradation, and (iv) its stabilization of the nanocarrier system, making it a favorable excipient in delivery of active compounds. Nevertheless, a majority of the TPGS-based targeted nanomedicines are still at the laboratory scale with limited preclinical evaluation. This development can be accelerated by optimizing the industrial scale-up process and selecting appropriate models to minimize physiological variations between animals and humans. Nanoparticles often exhibit complex pharmacokinetics and biodistribution profiles that differ from conventional active compounds. Factors such as the particle size, surface charge, and protein corona formation can influence these properties. Unpredictable behavior in vivo can affect the therapeutic efficacy and safety of nanomedicines. Comprehensive preclinical safety studies and rigorous clinical trials are necessary to evaluate the biocompatibility and toxicity of nanoparticles. Additionally, advanced imaging techniques and computational modeling can be used to study and predict the behavior of nanoparticles in biological systems.

It is worth noting that the ideal stability range for TPGS lies within pH 4.5–7.5. Extremely acidic or alkaline conditions can lead to the hydrolysis of its ester linkages, resulting in instability and degradation [119]. While it is improbable that the production, handling, or ingredients of a food designed for special medical purposes would result in such circumstances [28], it is still important to take this concern into account. To the best of our knowledge, no study has been conducted to improve the stability of TPGS beyond its optimal pH range. Possible actions to address this issue include chemical modification of the TPGS structure to exert steric hindrance, thereby impeding the ability of a nucleophile to approach the bond and trigger a hydrolysis reaction. Before proposing a specific solution, it is crucial to study the role of each structural component of TPGS to identify which part of the molecule can be modified. The hydrophobic vitamin E segment facilitates membrane interaction and insertion, disrupting lipid packing within biological barriers such as the intestinal epithelium or cellular membranes, thus enhancing permeability [120]. The PEG chain provides hydrophilicity, steric stabilization, and improved solubility in aqueous environments, which collectively enhance nanoparticle dispersion and prevent aggregation [121,122]. The terminal hydroxyl group at the PEG end does not directly contribute to membrane penetration but serves as a chemical handle that can be selectively modified without significantly impairing TPGS’s permeation-enhancing properties. We propose several chemical modifications that can enhance stability. First, the conversion of ester bonds to ether bonds via deoxygenative reduction is a viable strategy [123]. Ether bonds are less susceptible to hydrolysis than ester bonds under both alkaline and acidic conditions, making them a strong alternative for improving the stability. Another approach involves altering the length of the PEG (polyethylene glycol) chain. Longer PEG chains provide greater steric hindrance, protecting ester bonds from hydrolysis by limiting water molecule access. However, extending the PEG chain may impact therapeutic efficacy, as shown by Zhao’s research which demonstrated that longer PEG chains were associated with a reduced anticancer effect [124]. Nevertheless, whether this observation applies to all types of drugs requires further investigation. Finally, crosslinking with high-molecular-weight polymers offers another promising strategy which can significantly increase steric hindrance, further protecting ester bonds from degradation. However, this approach may reduce solubility, potentially affecting the compound’s effectiveness in specific applications. These chemical modifications necessitate careful consideration to balance stability, solubility, and therapeutic activity effectively.

Therefore, a more comprehensive approach involving the development of diverse active compound-loaded TPGS-incorporated formulations and subsequent comparison is essential to identify the optimal TPGS-based nanocarrier for each active compound. Overall, standardized comparisons of TPGS-based nanocarriers are imperative to enhance the understanding of their impact on the bioavailability of a specific active compound.

## Figures and Tables

**Table 1:** Comparison of nanocarrier types: structure, drug loading, and stability.

Type of Nanocarrier	Characteristics		
	Structure	Drug Loading	Stability
Polymer-based			
Micelle	Amphiphilic block copolymer self-assembled into a core–shell structure	Hydrophobic drug loaded into the core	Sensitive to dilution and environmental changes
Polymersome	Vesicle composed of amphiphilic block copolymers	Can entrap both hydrophilic and hydrophobic drugs	Enhanced stability compared to liposome
Amorphous solid dispersion	Drug dispersed in a polymer matrix in an amorphous state	Improve solubility of poorly water-soluble drugs	Thermodynamic instable and may crystallize over time
Lipid-based			
Liposome	Phospholipid bilayer surrounding an aqueous core	Hydrophilic drug in core, hydrophobic drug in bilayer	Prone to fusion and leakage
Polymer lipid hybrid nanoparticle	Polymer core with a lipid shell	Can load both hydrophilic and hydrophobic drugs	Stable as it inherits advantages from both parental carriers
Niosome	Non-ionic surfactant vesicle with a bilayer structure	Hydrophilic drug within the core and hydrophobic drug between the bilayer	More stable than liposome
Solid lipid nanoparticle	Solid lipid core stabilized by surfactants	Can load both hydrophilic and hydrophobic drugs	Susceptible to lipid polymorphism-induced drug leakage
Lipid nanocapsule	Oily liquid core enclosed by a solid lipid-based shell	Lipophilic drug within the core	Highly stable
Self-emulsifying drug delivery system	Oil in water emulsion comprising a mixture of active compound, liquid oil, surfactant, and co-surfactant	Able to incorporate both hydrophilic and hydrophobic drugs	Thermodynamically stable
Nanocrystal			
Nanocrystal	Crystalline drug particles, stabilized by surfactants	Incorporate hydrophobic drug	Require stabilizer to enhance stability
Nanosuspension			
Nanosuspension	Nanodrug particles dispersed within aqueous or organic medium	Improve solubility of hydrophobic drug	Thermodynamically unstable

**Table 2:** Challenges in oral active compound formulation and the roles of TPGS.

Challenges in Oral Active Compound Formulation	Potential of TPGS as an Oral Active Compound Bioavailability Enhancer
Solubility: Many active compounds have poor water solubility, leading to low bioavailability when administered orally.	The amphiphilic nature of TPGS enables it to form hydrogen bonding with hydrophilic active compounds and engage in hydrophobic interaction with lipophilic active compounds, allowing it to effectively dissolve both types of active compounds, making it a potent solubility enhancer.
Stability: Active compounds can degrade due to factors such as moisture, heat, light, and pH variations in the gastrointestinal tract.	Encapsulation of active compound within TPGS coating effectively forms a protective shield around the active compound, blocking the access of degradative enzymes to the active pharmaceutical compound.
First-pass metabolism: The liver metabolizes many active compounds before they reach systemic circulation, significantly reducing their bioavailability.	TPGS-containing formulation strategies such as proactive compounds, enzyme inhibitors, etc., are used to bypass or reduce first-pass metabolism.
Permeability: Some active compounds have low permeability across the gastrointestinal membrane.	TPGS enhances active compound permeability by inhibiting P-gp through modulation of membrane fluidity and P-gp ATPase inhibition.
Bioavailability: The fraction of the administered active compound that reaches the systemic circulation in an active form can be very low for some active compounds.	Enhancing solubility, using permeation enhancers, and employing active compound delivery systems like nanoparticles can improve bioavailability.

ATPase: Adenosine triphosphatase; etc: et cetera; P-gp: P-glycoprotein; TPGS: D-α-tocopheryl polyethylene glycol 1000 succinate.

**Table 3:** Mechanism of action, advantages, and disadvantages of several common absorption enhancers.

Common Absorption Enhancer	Mechanism of Action	Advantage(s)	Disadvantage(s)
TPGS	Solubilizes drug and inhibit P-gp efflux	Tailorable chemical modifications to meet the specific needs of drug delivery systems	Non-specific P-gp inhibition
Chitosan	Increases permeability of intestinal wall	Non-toxic and biodegradable	pH-sensitive
Bile salt	Reduces surface tension and increases drug solubility	Biocompatible and readily metabolized by the body	High concentration can cause significant membrane damage and local irritation
Chelating agent such as ethylene glycol tetraacetic acid and ethylene diamine tetraacetic acid	Bind to calcium ions, disrupting cell–cell contacts	Synergistic with other enhancers	- Compromise drug or excipient stability due to the formation of complexes with metal ions present in the formulation - Disrupt local calcium-dependent physiological processes due to excessive calcium chelation
Fatty acid and derivatives such as sodium caprate and salcaprozate sodium	Disrupt cellular tight junctions	Biocompatible and protects drug against degradation	Dose-dependent irritation and less useful for lipophilic drugs

P-gp: P-glycoprotein; TPGS: D-α-tocopheryl polyethylene glycol 1000 succinate.

**Table 4:** Characteristics and pharmacokinetics improvement of TPGS nanocarrier system.

Type of Nanocarrier	Study	Co-Ingredient	Preparation Method	Active Compound	Particle Size (nm)	Zeta Potential (mV)	Polydispersity Index	Encapsulation Efficiency (%)	Experimental Model	Dosage (mg/kg)	AUC (Free Form in Bracket)	AUC Increment as Compared to Free Form (-Fold)	T_1/2_ (h) (Free Form in Bracket)	T_max_ (h) (Free Form in Bracket)	C_max_ (Free Form in Bracket)	Storage Stability (Month)	Reference
Micelle	1	Carboxymethyl chitosan (CMCS) and rhein	Sonication and dialysis	Paclitaxel	193.0 ± 1.0	–32.1 ± 0.4	0.126 ± 0.004	85.53 ± 3.36	Sprague Dawley rat	20	10.50 ± 1.96 (0.57 ± 0.23) µg/mL × h	18.420	N/A	3.20 ± 0.45 (1.60 ± 0.89)	1.14 ± 0.61 (0.13 ± 0.06) µg/mL	N/A	[36]
	2	Poly(ε-caprolactone) (PCL)	Solvent emulsification–evaporation	Darunavir (DRV)	173.74 ± 8.01	−21.5 ± 0.212	0.218 ± 0.01	82.32 ± 4.18	Sprague Dawley rat	20	22,3031.61 ± 11.4 (65,248.79 ± 8.19) ng/mL × h	3.42	N/A	12.14 ± 1.45 (5.07 ± 0.30)	15,645.65 ± 2.03 (8467.36 ± 4.78) ng/mL	N/A	[37]
	3	Galactosamine, polyethylene glycol (PEG) and polylactic acid (PLA)	Thin-film dispersion	Curcumin	100.02 ± 0.55	−8.77 ± 0.73	0.127 ± 0.01	84.31 ± 0.11	Female Wistar rat	50	2. 20 ± 0.40 µg/mL × h	N/A	N/A	1.17 ± 0.65	245.87 ± 0.17 ng/mL	N/A	[38]
	4	Phospholipid, sodium cholate and polyvinylpyrrolidone K30 (PVP K30)	Thin-film dispersion	Zingerone	50.62 ± 0.25	−28.07 ± 0.33	0.168 ± 0.006	94.71 ± 2.02	Male Sprague Dawley rat	300	44.80 ± 0.78 (8.79 ± 0.21) µg/mL × h	5.10	3.22 ± 0.24 (1.7660:09)	2 (0.5)	8.86 ± 0.03 (4.2860:07) µg/mL	1	[39]
	5	NO	Thin-film hydration	Lopinavir	91.71	−24.8	0.129	99.36 ± 1.06	New Zealand rabbit	20	639.22 ± 28.39 (201.33 ± 17.68) ng/mL × h	3.17	15.57 ± 4.80 (4.66 ± 0.27)	1.54 ± 0.033 (1.55 ± 0.03)	165.573 ± 3.05 (49.05 ± 2.53) ng/mL	6	[40]
	6	Pluronic F127	Thin-film hydration	Glycyrrhizic acid (GL)	27.41 ± 4.90	−5.92 ± 0.68	0.19 ± 0.07	95.38 ± 3.22	Male Sprague Dawley rat	50	79.66 ± 10.83 (44.20 ± 15.25) mg/L × h	1.80	4.83 ± 2.06 (4.58 ± 1.79)	6.33 ± 0.82 (8.00 ± 0.00)	8.16 ± 2.19 (4.83 ± 1.41) mg/L	N/A	[41]
	7	Soluplus, borneol	Thin-film hydration	Aripiprazole (ARP)	52.37 ± 1.35	−4.44 ± 0.32	0.07 ± 0.02	98.48 ± 2.39	Male CD1 mice	0.2	14,181 ± 551 (8463 ± 503) ng/mL × h	1.68	17.50 ± 0.83 (13.40 ± 0.75)	4 (4)	738 ± 28.7 (437 ± 26.4) ng/mL	3	[42]
	8	Gallic acid and chitosan	Ultrasonic emulsification	Paclitaxel	134.9 ± 10.2	34.8 ± 1.3	0.172 ± 0.130	80 ± 3	Male Sprague Dawley rat	11	2528 ± 294 (665 ± 129) ng/mL × h	3.80	19.8 ± 10.4 (12.7 ± 3.4)	3.0 ± 0 (1.5 ± 0)	308 ± 103 (78 ± 34) ng/mL	N/A	[43]
	9	Poly (ethylene glycol)-poly (ε-caprolactone) (PEG-PCL)	Solvent injection	6-gingerol	73.24 ± 2.84	−2.74 ± 0.92	0.129 ± 0.03	79.68	Male Sprague Dawley rat	250	497.36 ± 48.11 (89.88 ± 22.84) µg/mL × min	5.53	N/A	0.25 (0.083)	9.55 ± 1.12 (2.66 ± 0.19) µg/mL	N/A	[44]
	10	NO	Thin-film hydration	Curcumin	12.3 ± 0.1	N/A	0.17	N/A	Male Wistar rat	150	3461.48 ± 102.47 (529.49 ± 22.32) ng/mL × h	6.54	3.16 ± 0.78 (0.35 ± 0.07)	2 (0)	794.97 ± 43.94 (311.42 ± 15.51) ng/mL	0.25	[45]
	11	Phospholipids, sodium cholate and polyvinylpyrrolidone K30 (PVP K30)	Thin-film dispersion	Myricetin (MRC)	26.42 ± 0.89	−23.23 ± 0.79	0.135 ± 0.017	93.8 ± 1.18	Male Sprague Dawley rat	300	19.648 ± 2.779 (5.535 ± 0.729) µg/mL × h	3.55	9.208 ± 0.233 (8.101 ± 0.0534)	7 ± 0 (7 ± 0)	1.630 ± 0.112 (0.584 ± 0.052) µg/mL	N/A	[46]
	12	NO	Solvent dispersion	Hesperetin	26.19 ± 0.05	N/A	0.257 ± 0.024	N/A	Female Sprague Dawley rat	100	53.01 ± 4.39 (3.28 ± 0.68) µg/mL × h	16.16	18.45 ± 17.86 (0.39 ± 0.00)	0.39 ± 0.10 (0.61 ± 0.24)	20.67 ± 8.27 (2.64 ± 0.76) µg/mL	N/A	[47]
Polymersome	13	Folic acid (FA), Pluronic F127 and polylactic acid (PLA)	Direct injection and dialysis	Paclitaxel	108.53	N/A	0.34	15.53	Sprague Dawley rat	0.15	3737.14 ± 631.58 (559.18 ± 113.90) ng/mL × h	6.68	N/A	3.20 ± 1.34 (1.40 ± 0.55)	228.31 ± 59.46 (51.72 ± 17.52) ng/mL	N/A	[48]
Amorphous solid dispersion	14	Kollidon CLSF	Solvent Evaporation	Curcumin	N/A	N/A	N/A	N/A	Sprague Dawley rat	100	1186.0 ± 59.7 (724.3 ± 11.0) ng/mL × h	1.64	N/A	0.33 (0.50)	625.20 ± 66.98 (227.28 ± 22.28) ng/mL	2	[49]
Liposome	15	Lecithin and cholesterol	Thin-film hydration	Bisdemethoxycurcumin (BDMC)	75.98 ± 5.46	− 38.21 ± 0.29	0.35 ± 0.016	96.98 ± 0.17	Male Sprague Dawley rat	100	6.06 ± 1.18 (0.58 ± 0.18) mg/L × h	10.45	5.7 ± 0.98 (1.512 ± 0.34)	0.5 ± 0 (0.17 ± 0)	1.38 ± 0.37 (0.4 ± 0.08) mg/L	N/A	[50]
	16	Phospholipid, cholesterol, sodium cholate, isopropyl myristate (IPM)	Thin-film dispersion	6-shogaol	23.50 ± 0.09	−45.40 ± 2.2	0.140 ± 0.003	95.18 ± 0.28	Male Sprague Dawley rat	200	2220.41 ± 24.21 (382.80 ± 47.24) µg/mL × min	5.80	6.08 (3.25)	1 (0.5)	5.09 ± 0.24 (2.23 ± 0.16) µg/mL	N/A	[51]
	17	Zein and lecithin	Phase separation	Rapamycin	190.31 ± 9.02	−19.71 ± 1.125	0.256 ± 0.029	86.64 ± 2.43	Male Sprague Dawley rat	20	18,021.44 ± 1300.29 (7313.65 ± 934.83) ng/mL × h	2.46	55.95 ± 3.39 (34.12 ± 2.65)	2.00 ± 0.64 (4.00 ± 0.36)	1052.05 ± 173.11 (516.80 ± 33.05) ng/mL	N/A	[52]
	18	Lecithin and cholesterol	Thin-film dispersion	Syringic acid (SA)	40.01 ± 0.48	− 38.50 ± 0.05	0.22 ± 0.02	96.48 ± 0.76	Male Sprague Dawley rat	25	338.08 ± 3.65 (120.58 ± 2.92) µg/mL × min	2.80	0.83 (0.29)	0.133 (0.133)	4.50 ± 0.04 (4.50 ± 0.04) µg/mL	1	[53]
Polymer Lipid Hybrid Nanoparticle	19	Polycaprolactone, phospholipon 90 G, poloxamer	Single-step nanoprecipitation	Exemestane	136.37 ± 3.27	N/A	0.110 ± 0.013	88.56 ± 2.15	Female Wistar rat	20	2444.33 ± 204.66 (520.29 ± 122.29) ng/mL × h	4.70	22.211 ± 0.754 (11.898 ± 1.025) h	4 (2) h	312.8 ± 18.21 (76.81 ± 9.95) ng/mL	N/A	[54]
	20	Poly(lactic-co-glycolic acid) (PLGA), glyceryl monostearate, soybean lecithin, poly(methyl vinyl ether-co-maleic anhydride) (PVMMA) and poloxamer 188	Emulsification-solvent evaporation	Cabazitaxel (CTX)	192.2 ± 4.0	−35.65 ± 2.46	0.241 ± 0.015	92.1 ± 3.7	Male Sprague Dawley rat	10	615.77 ± 296.87 (127.78 ± 76.77) µg/L × h	4.82	N/A	1.08 ± 0.22 (0.30 ± 0.11)	209.94 ± 76.18 (53.53 ± 34.97) µg/L	N/A	[55]
Niosome	21	Span 60 or Span 40 and cholesterol	Thin-film hydration	Pyrazolopyrimidine	138.9 ± 16.8	−45.1 ± 8.9	0.21 ± 0.03	90.6 ± 5.1	Male Wistar albino rat	20	126.6 ± 16.3 (28.1 ± 8.2) mcg/mL × h	4.51	N/A	N/A	9.2 ± 2.1 (2.52 ± 0.71) mcg/ml	N/A	[56]
Solid Lipid Nanoparticle	22	Glycerylmonooleate (GMO)	Emulsification and homogenization	Naringenin	256	−10.6 ± 2.1	N/A	72.70 ± 0.80	Male Wistar rat	20	15.0 ± 4.0 (2.0 ± 0.5) µg/mL × h	7.5	2.3 (2.3)	N/A	4.3 ± 1.2 (0.3 ± 0.1) µg/mL	N/A	[57]
	23	Glyceryl monostearate (GMS) and Poloxamer 188	High-speed homogenization followed by ultrasonication	Asenapine maleate (AM)	114.3 ± 3.5	−12.9 ± 3.8	0.188 ± 0.010	84.10 ± 2.90	Female Sprague Dawley rat	1.033	27,460.50 ± 151.90 (547.12 ± 28.47) ng/mL × h	50.19	7.61 ± 1.19 (4.13 ± 0.58)	8.00 ± 0.21 (1.00 ± 0.31)	1396.44 ± 116.81 (67.19 ± 8.40) ng/mL	3	[58]
Lipid nanocapsule	24	Maisine™ 35-1	Antisolvent precipitation	Curcumin	190	N/A	0.24	51.06 ± 7.27	Male Sprague Dawley rat	100	1174.42 ± 567.99 (95.64 ± 34.77) ng/mL × h	12.28	N/A	2.0 ± 0.1 (0.5 ± 0.1)	171.23 ± 67.88 (30.56 ± 10.22) ng/mL	3	[59]
Self-emulsifying drug delivery system (SEDDS)	25	Polyethylene glycol 200 (PEG 200) and pumpkin seed oil (PSO)	Probe sonication	Quercetin (QU)	320 ± 34.3	−28.6 ± 4.1	0.37 ± 0.07	N/A	Male Wistar rat	25	2286 ± 500.1 (1525.7 ± 378.8) µg/L × h	1.50	N/A	0.5 ± 0.0 (0.83 ± 0.26)	491.3 ± 172.2 (163.2 ± 74) µg/L	0.5	[60]
	26	Peceol, Solutol HS15 (polyethylene glycol-15-hydroxystearate) and calcium silicate	High-pressure homogenization and spray drying	Rivaroxaban	241.2 ± 26.0	N/A	0.184 ± 0.079	N/A	Male Sprague Dawley rat	0.5	554.11 ± 130.83 (156.49 ± 76.67) ng/mL × h	3.54	1.32 ± 0.45 (2.88 ± 3.83)	0.63 ± 0.14 (0.46 ± 0.19)	320.92 ± 96.91 (80.45 ± 14.85) ng/mL	N/A	[61]
	27	Vitamin E, Labrafa, Capryol^®^ 90 and Gelucire^®^	Mixing and Spray-drying	Paclitaxel	30.00 ± 2.00	17.38 ± 2.88	0.198 ± 0.050	95.63 ± 3.36	Female Sprague Dawley rat	20	16,071.00 ± 2580.00 (2657.00 ± 208.80) ng/mL × h	6.05	N/A	4.46 ± 0.59 (1.14 ± 0.16)	1627.00 ± 281.50 (403.90 ± 78.02) ng/mL	6	[62]
Nanocrystal	28	Sodium dodecyl sulfate (SDS)	High-pressure homogenization	Andrographolide (ADR)	604.6 ± 5.7	N/A	0.198 ± 0.038	N/A	Male Sprague Dawley rat	50	3.428 ± 0.789 (1.286 ± 0.218) mg/L × h	2.67	8.246 ± 2.915 (2.809 ± 0.232) h	0.517 ± 0.32 (0.717 ± 0.415)5 h	0.602 ± 0.146 (0.229 ± 0.082) mg/L	N/A	[63]
	29	NO			573.2 ± 3.9	46.27 ± 0.25	0.223 ± 0.024	N/A			4.602 ± 0.969 (1.286 ± 0.218) mg/L × h	3.58	8.145 ± 1.227 (2.809 ± 0.232) h	0.850 ± 0.224 (0.717 ± 0.415) h	0.791 ± 0.330 (0.229 ± 0.082) mg/L	N/A	[64]
Nanosuspension	30	NO	Tetragonal zirconia polycrystal (TZP) grinding method	Quercetin (QU)	182.1 ± 1.5	N/A	0.21 ± 0.02	N/A	Male Sprague Dawley rat	50	387.09 ± 60.28 (89.93 ± 38.42) ng/mL × h	4.30	N/A	0.61 ± 0.71 (0.53 ± 0.34)	52.68 ± 16.87 (16.73 ± 6.33) ng/mL	1	[65]
	31	Polyvinyl alcohol (PVA)	Antisolvent precipitation	Ticagrelor (TCG)	233 ± 2	–8.9 ± 0.5	0.173 ± 0.022	N/A	Male Sprague Dawley rat	10	2112.2 ± 268.1 (1026.5 ± 463.7) ng/mL × h	2.06	4.4 ± 2.3 (4.3 ± 1.8)	0.7 ± 0.5 (1.8 ± 1.3)	571.3 ± 144.9 (189.0 ± 54.7) ng/mL	N/A	[66]

AUC: Area under curve; C_max_: maximal concentration; N/A: data are not available; T_1/2_: half-life; T_max_: time taken for an active compound to reach the maximum concentration; TPGS: D-α-tocopheryl polyethylene glycol 1000 succinate.
